# The Impact of Chronic Pulmonary Aspergillosis Co-infection on the Health-Related Quality of Life of Patients with Pulmonary Tuberculosis in Uganda

**DOI:** 10.21203/rs.3.rs-2389854/v1

**Published:** 2023-01-13

**Authors:** Martha Namusobya, Felix Bongomin, John Mukisa, Charles Batte, William Kane Olwit, Joshua Rhein, Christine Sekaggya-Wiltshire, Shailendra Prasad

**Affiliations:** Makerere University CHS: Makerere University College of Health Sciences; Gulu University Faculty of Medicine; Makerere University Faculty of Medicine: Makerere University College of Health Sciences; Makerere University Faculty of Medicine: Makerere University College of Health Sciences; Makerere University Medical School: Makerere University College of Health Sciences; University of Minnesota Department of Medicine; Makerere University Infectious Diseases Institute; University of Minnesota Department of Medicine

**Keywords:** Pulmonary Tuberculosis, Persistent symptoms, Chronic Pulmonary Aspergillosis, Quality of Life, St. George Respiratory Questionnaire, Uganda

## Abstract

**Background:**

Both pulmonary tuberculosis (PTB) and chronic pulmonary aspergillosis (CPA) significantly affect health-related quality of life (HR-QoL). We aimed to determine the impact of CPA co-infection on the HR-QoL of Ugandans with PTB.

**Methods:**

We conducted a prospective study among participants with PTB with persistent pulmonary symptoms after 2 months of anti-TB treatment at Mulago Hospital, Kampala, Uganda between July 2020 and June 2021. HR-QoL was assessed using St. George Respiratory Questionnaire (SGRQ) at enrollment and at the end of PTB treatment (4 months apart). SGRQ scores range from 0 to 100, with higher score representing a poorer HR-QoL.

**Results:**

Of the 162 participants enrolled, 32 (19.8%) had CPA + PTB and 130 (80.2%) had PTB only. The baseline characteristics of the two groups were comparable. Regarding overall health, a higher proportion of the PTB only group rated their HR-QoL as “very good” compared to those who had both TB and CPA (68 (54.0%) *versus* 8 (25.8%)). At enrollment, both groups had comparable median SGRQ scores. However, at follow up, the PTB only group had statistically significantly better SGRQ scores (interquartile range); symptoms (0 (0 – 12.4) *versus* 14.4 (0 – 42.9), p < 0.001), activity ((0 (0 – 17.1) *versus* 12.2 (0 – 35.5), p = .03), impact (0 (0 – 4.0) *versus* 3.1 (0 – 22.5), p = 0.004), and total scores ((0 (0 – 8.5) *versus* 7.6 (0 – 27.4), p = 0.005).

**Conclusion:**

CPA co-infection impairs HR-QoL of people with PTB. Active screening and management of CPA in patients with PTB is recommended to improve HR-QoL of these individuals.

## Introduction

Tuberculosis (TB) is a global public health problem and the leading cause of death from a single infectious agent (1). Pulmonary TB (PTB) is the most common form, accounting for over 60% of the disease (1). The hallmark of PTB sequalae is lung impairment through destruction of its architecture, including cavitation, fibrosis and bronchiectasis leading to reduced pulmonary function (2). An estimated 18–87% of patients with PTB experience lung impairment following microbiological cure (2) and higher mortality rates of up to 3-fold higher than in the general population (3).

The true burden of post-TB sequalae in our Ugandan setting is without doubt underreported due to paucity of clinical, research, and advocacy data. Post-TB sequalae may manifest as structural complications including bronchiectasis, bronchiolitis, residual cavitation, or chronic obstructive pulmonary disease (COPD); infectious complications including chronic pulmonary aspergillosis (CPA); and psychosocial complications (4). Overall, post-TB complications are diverse with a resultant effect of significant impairment on the health-related quality of life (HR-QoL) (4).

PTB sequalae manifest through a decline in HR-QoL (3). Studies done in South Africa and Uganda show significant decline in HR- QoL among PTB patients (5, 6, 7). It is important to evaluate the psychosocial well-being and HR-QoL of patients (8, 9). Measurements of HR-QoL are increasingly proving to be key and are important indicators in day-to-day patient care, research, and even in programmatic monitoring and evaluation of populations (8, 9).

Patients with PTB are at an increased risk of CPA (10, 11), and superimposed CPA leads to poorer PTB outcomes (11, 12). We sought to investigate and compare HR-QoL of participants treated for PTB and those whose PTB was complicated with CPA to understand if CPA worsened HR-QoL in this patient population.

## Methods

### Study design

This was a nested sub study (13). We conducted a prospective observational study at the National TB control center of Mulago National Referral Hospital (MNRH), Kampala, Uganda between 1st July 2020 and 30th June 2021.

### Study Setting

The TB Unit at MNRH serves as the national TB treatment center in Uganda. The unit uses a mixed model of care, whereby, 1) very sick patients are hospitalized at the start of their TB treatment until clinically stable, and 2) outpatient care where patients continue treatment from the community under supervision. The unit manages about 1,500 TB patients annually, making it the largest treatment center in the country.

### Study Population

We enrolled all eligible patients 18 years and older with microbiologically confirmed drug sensitive PTB (DS-PTB) using GeneXpert MTB/RIF and persisting pulmonary and/or systemic symptoms despite 2 months of standard anti-TB treatment. Patients on second line anti-TB regimens, pregnant women, critically ill patients, and those with extra-pulmonary TB were excluded.

### Study Procedure

HR-QoL was assessed using the St. George Respiratory Questionnaire (SGRQ). QoL data was collected at 2 time points: at the baseline/enrollment visit, concurrently with the CPA diagnosis data, then at the PTB treatment completion visit (4 months later), which also doubled as the study follow up visit.

### St. George Respiratory Questionnaire

The SGRQ was the tool used to collect HR-QoL data for this study. The SGRQ is a 50 item survey designed to measure the impact of lung disease on overall mental health and wellbeing. It contains three components: symptoms, activity level, and the impact of lung health on daily life. The total score, calculated for all items, provides a global view of the patient’s respiratory health. It is scaled from 0 (optimal) to 100 (worst); responses are used to produce a score for each component and an aggregate score. It has been successfully used previously to evaluate quality of life among pulmonary TB patients (14, 15). Notably, the SGRQ is validated for CPA (16, 17, 18) and validated in Uganda where our study was based (19). SGRQ HR-QoL measures were interpreted as described in earlier literature (20, 21).

### Data analysis

Baseline characteristics of the study population were summarized using percentages for categorical data and compared using chi square tests and Fisher exact tests.

Median interquartile ranges were calculated for continuous variables. The quality of life as measured using the SGRQ was calculated for all participants based on the SGRQ manual (22). Baseline and follow up median QOL scores were compared using the Wilcoxon rank sum tests in the PTB alone and PTB + CPA groups. Box plots considering the symptoms, activity, impacts and total scores were drawn for visualization of the data. For all analyses, a P-value of less than or equal to 0.05 was considered as statistically significant. All data analysis was conducted in STATA V.14 (StataCorp, College Station, Texas).

## Results

### Baseline clinical characteristics of the participants

Of the 162 participants enrolled, 97 (60.0%) were male with a median age for all participants of 30 (IQR: 25 – 40) years. Forty-eight (29.6%) participants were living with HIV and 15 (9.3%) were previously treated for PTB.

Overall, 32/162 (19.8%) participants had CPA. Of the 32 participants with CPA, 3/32 (9.3%) were commenced on antifungal therapy. The antifungal therapy consisted of a standard guideline recommended dose of itraconazole of 200mg twice daily. The clinical characteristics of patients with TB alone and those with TB + CPA were comparable. However, a slightly higher of those with TB alone had chest pain (90.6% versus 93.1%, p = 0.016), Table 1.

### Overall Self-rated Health Status

The difference in overall health, both at baseline and follow up between the participants who had both PTB and CPA (p = 0.424) were not statistically significantly different compared to those who had PTB only (0.342). However, a higher proportion of participants in the PTB only group reported favorable outcomes in their overall health at follow-up, with 68 (54.0%) participants in the PTB-only group reporting a “very good” compared to only 8 (25.8%) who had both TB and CPA, Table 2.

### Quality Of Life Across Domains Of St. George’s Respiratory Questionnaire

**Figure 1:**
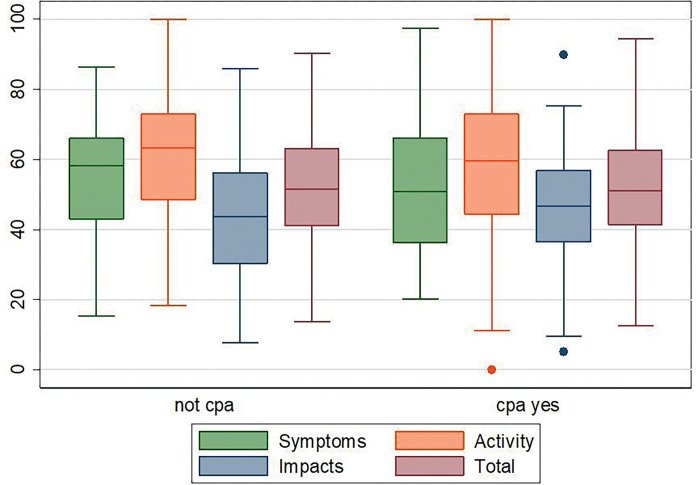
Box plots of the SGRQ scores by the CPA status. The top and bottom of the boxes represent the 25^th^ and 75^th^ percentile while the horizontal line represents the median and the dots represent the outliers. Baseline scores by CPA status

**Figure 2:**
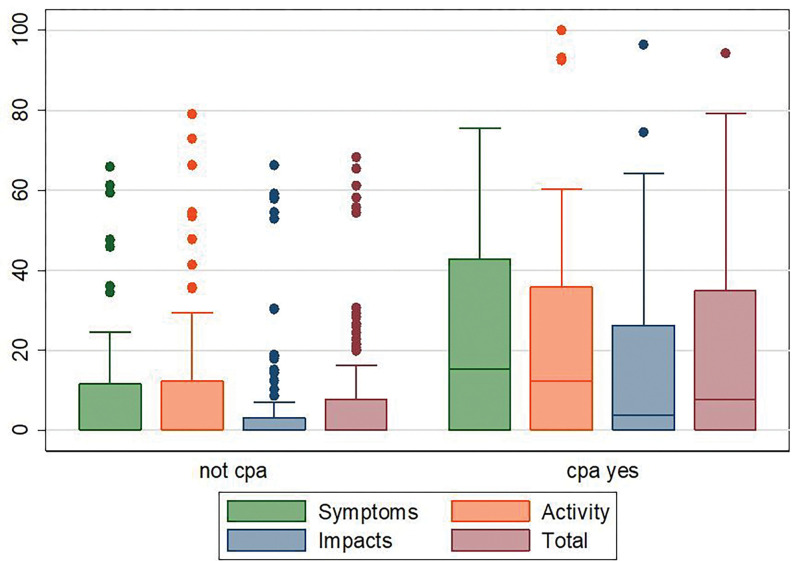
Box plots of the SGRQ scores by the CPA status. The top and bottom of the boxes represent the 25^th^ and 75^th^ percentile while the horizontal line represents the median and the dots represent the outliers. Follow up scores by CPA status

At enrollment (baseline, 2 months of anti-TB therapy, no antifungal therapy), both groups had comparable median quality of life scores; symptoms (55.4 (42.9 – 66.0) *versus* 53.3 (37.6– 66.8, p = 0.987), activity (60.3 (47.7 – 72.8) *versus* 59.5 (47.5 – 72.8), p = 0.542), impact (43.6 (30.1 – 56.2) *versus* 46.6 (37.7 – 56.7), p = 0.358), and total scores (51.0 (40.0 – 63.1) *versus* 51.7 (42.9 – 62.5), p = 0.699), Table 3, Fig. 1. However, at follow up (end of TB therapy), participants with TB alone compared to those with had statistically significantly better median (IQR) quality of life scores; symptoms (0 (0 – 12.4) *versus* 14.4 (0 – 42.9), p < 0.001), activity ((0 (0 – 17.1) *versus* 12.2 (0 – 35.5), p = .03), impact (0 (0 – 4.0) *versus* 3.1 (0 – 22.5), p = 0.004), and total scores ((0 (0 – 8.5) *versus* 7.6 (0 – 27.4), p = 0.005), Table 4, Fig. 2.

## Discussion

In this prospective cohort study, we showed that participants with PTB only had statistically significantly better SGRQ scores compared to those with PTB and CPA co-infection. Our findings are consistent with previous literature that showed that CPA significantly affects HR-QoL, particularly for those who have not been initiated on anti-fungal therapy (16). Of the 32 participants with CPA in our study, only 3 received anti-fungal therapy. This could explain the poor HR-QoL experienced by the CPA/PTB group.

The WHO promotes patient involvement in healthcare decisions because this allows them to be more proactive in their management and ultimately more able to adhere with treatment plans (5, 23). The use of disease-specific health status questionnaires helps to discriminate between different levels of disease severity. Patient experience can be assessed using a patient-reported outcome measure (PROM). The SGRQ as a form of PROM has already been successfully validated in Uganda (19).

At enrollment, we observed that participants were quite ill, and therefore we recorded the highest SGRQ scores at this point of the study. The scores then improved markedly in the different HR-QoL domains at the follow up visit which coincided with end of anti-TB therapy. This was very likely a reflection of the known efficacy of anti-TB therapy and adherence to this treatment by the participants.

While studies that measured HR-QoL among TB patients are still few in Africa (24), there is some precedence already set by another study like ours that investigated both PTB and CPA and their effects on HR-QoL (25). Such research will go a long way in improving management and outcomes for these patients.

This finding of improvement in HR-QoL due to anti-TB therapy accords with findings from similar studies (26, 27, 28, 29). Thus from a programmatic perspective, ensuring adherence to treatment and retention in care is important for improvement in HR-QoL. For our study, only 9.3% of participants diagnosed with CPA were able to access treatment since itraconazole remains expensive in our setting and not included on the Ministry of Health essential medicines list.

Both at baseline and follow up, there were no significant differences (p = 0.424, 0.342) in overall health between the group that had PTB only and those that had both CPA and PTB. This may be explained with the fact that at baseline, both groups were ill, and therefore overall health was poor for both. At follow up, improvement due to effective anti-TB therapy likely, and temporarily, masked underlying CPA disease (whose genesis is more gradual and insidious) by improving lung inflammation, cavity size, and pleural disease hence giving symptom relief.

In comparing the SGRQ scores by CPA status at baseline, there was no significant difference in scores between the TB/CPA group versus the TB only group. This would still be explained by illness in both groups at baseline. Conversely, at follow up, a statistically significant difference arises in SGRQ scores between the two groups. This could possibly be due to the persistence of symptoms in the group with CPA, as has been reported in previous studies (16, 25), and especially so because majority of CPA positive participants could not afford anti-fungal therapy.

This study had some limitations. This was a single center study, involving mainly patients from the central region of Uganda and may not be representative of other African populations, since social demographics and support systems that could influence HRQOL may differ across sites. Future multicenter studies are recommended.

In conclusion, HR-QoL- based disease appraisals in resource-limited settings are important instruments to grasp health outcomes and provide focused and empirically informed ways to manage care and treatment better (25). HR-QoL among PTB patients improves with anti-TB therapy, however co-infection with CPA negatively impacts their HR-QoL. Therefore, programmatic approaches to screen, diagnose, and treat CPA co-infection among PTB patients will improve QoL and general well-being in this population.

## Figures and Tables

**Table 1:** Baseline Clinical Characteristics

Characteristics	All, (%)	CPA+PTB n = 32	PTB alone n(%) n = 130	P-value
**Shortness of breath**	67 (41.4)	10 (27.8)	57 (45.2)	0.061
**Weight loss**	84 (51.8)	18 (56.3)	66 (50.8)	0.900
**Fatigue**				
yes	75 (46.3)	15 (46.9)	60 (46.1)	0.899
**Fever**				
yes	31 (19.1)	5 (15.6)	26 (20.0)	0.669
**Loss of appetite**				
yes	59 (36.4)	11 (34.4)	48 (36.9)	0.663
**Shortness of breath**				
yes	67 (41.4)	9 (28.1)	58 (44.6)	0.061
**Hemoptysis**				
Yes	20 (12.4)	7 (21.9)	13 (10.0)	0.142
**Night sweats**				
Yes	105 (64.8)	18(56.3)	87 (66.9)	0.086
**Chest pain**				
Yes	150 (92.6)	29 (90.6)	121 (93.1)	0.016
**Wheezing**				
Yes	15 (9.3)	12 (9.2)	3 (9.4)	0.828

**Table 2:** Comparison of Overall Health between the TB group and the TB + CPA group.

Status	CPA+TB		TB alone	
	Baseline n (%).	Follow -up n (%).	Baseline n (%).	Follow –up n (%).
Very good	0 (0)	8 (25.8)	0 (0)	68 (54.0)
Good	6 (18.8)	9 (29.0)	22 (16.9)	41 (32.5)
Fair	21 (65.6)	11 (35.5)	84 (64.6)	11 (8.7)
Poor	5 (15.6)	1(3.2)	23 (17.7)	3(2.4)
Very poor	0(0.0)	2 (6.4)	1 (0.8)	3(2.4)
P-value	0.424		0.342	

**Table 3:** **Comparing the SGRQ scores by CPA status** Baseline, n = 162

Characteristic	TB alone	TB + CPA,	P value*
Symptoms, median, interquartile range	55.37 (42.85–66.03)	53.25(37.62–66.82)	0.987
Activities, median, interquartile range	60.26(47.68–72.82)	59.46 (47.46–72.82)	0.542
Impacts median, interquartile range	43.62(30.08–56.17)	46.61 (37.66–56.73)	0.358
Total score (median, interquartile range)	50.97 (39.9–63.05)	51.65(42.94–62.52)	0.699

* based on Wilcoxon rank sum test

**Table 4:** **Comparing the SGRQ scores by CPA status** Follow up, n = 157

Characteristic	TB alone	TB + CPA	P value*
Symptoms, median, interquartile range	0 (0–12.39)	14.43 (0–42.85)	< 0.001
Activities, median, interquartile range	0 (0–17.12)	12.17(0–35.47)	0.029
Impacts median, interquartile range	0 (0–3.97)	3.06(0–22.53)	0.004
Total score (median, interquartile range)	0 (0–8.48)	7.57(0–27.43)	0.005

* based on Wilcoxon rank sum test
